# Cystadénome séreux du pancréas associe à une hétérotopie pancréatique

**DOI:** 10.11604/pamj.2016.23.94.8533

**Published:** 2016-03-15

**Authors:** Hedfi Mohamed, Belghachem Dorra, Bouhafa Hela, Abdelhedi Cherif, Sridi Azza, Sassi Karim, Bellil Khadija, Chouchene Adnen

**Affiliations:** 1 Service de Chirurgie Générale Hôpital Des FSI La Marsa, Tunisie; 2 Service Anatomie Pathologique Hôpital des FSI La Marsa, Tunisie

**Keywords:** Pancréas, cystadénome, chirurgie, hétérotopie, Pancreas, cystadenoma, surgery, heterotopy

## Abstract

Les hétérotopies pancréatiques (HP) sont rares. Elles peuvent se voir à tout âge avec une légère prédominance masculine. ces lésions sont le plus souvent asymptomatiques, de découverte fortuite lors d'une endoscopie digestive haute ou basse ou lors de l'examen anatomopathologique d'un organe réséqué pour d'autres motifs, et peuvent être isolée ou associée à une pathologie digestive. Nous rapportons, à travers notre observation, l'association d'une HP à un cystadénome séreux du pancréas découverte lors de l'exploration des douleurs épigastriques isolées. A travers cette observation nous nous proposons d’étudier les particularités cliniques et histologiques de cette pathologie rare.

## Abstract (en)

Pancreatic heterotopias (HP) are rare. They can occur at any age with a slight male predominance. These lesions are usually asymptomatic and they are often found incidentally during upper or lower GI endoscopy or during the anatomo-pathological examination of an organ which was resected for other reasons; they can be isolated or associated with a digestive pathology. We report, through observation, the association of HP with serous cystadenoma of the pancreas discovered during examinations to identify the etiology of isolated abdominal pain. The aim of this study is to analyse clinical and histological features of this rare pathology.

## Patient et observation

**Figure 1:**
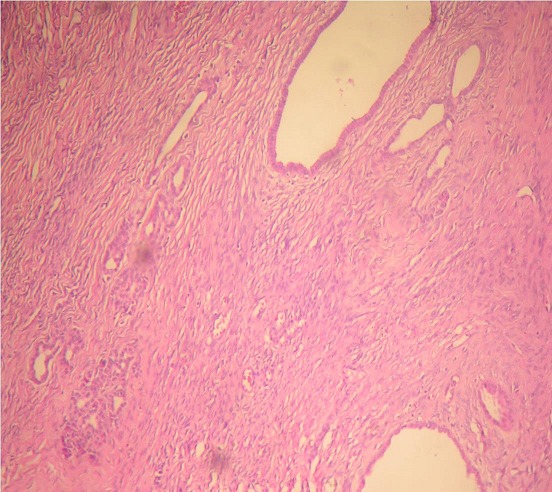
Strucutures canalaires associées à quelques acini (en fenêtre) (HEx200)

**Figure 2:**
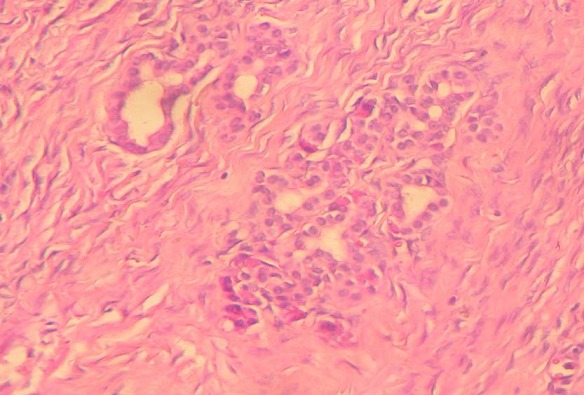
Strucutures canalaires

**Figure 3:**
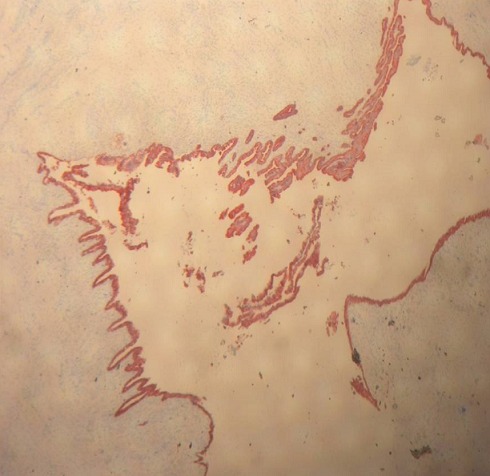
IHC: immunoréactivité des canaux au CK 19

**Figure 4:**
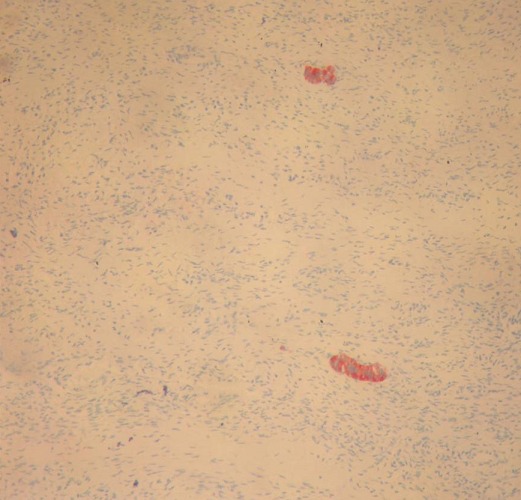
Immunoréactivité des ilots de Langerhans au synaptophysine

**Figure 5:**
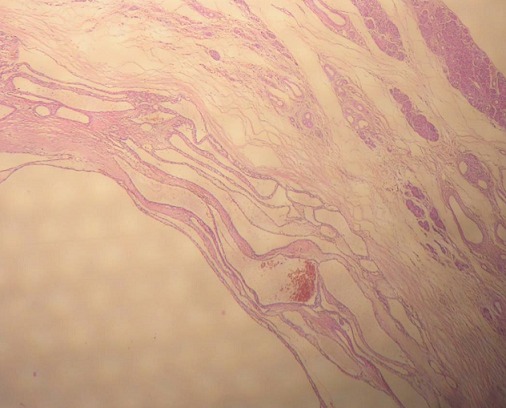
Cystadénome séreux (HEx100)

**Figure 6:**
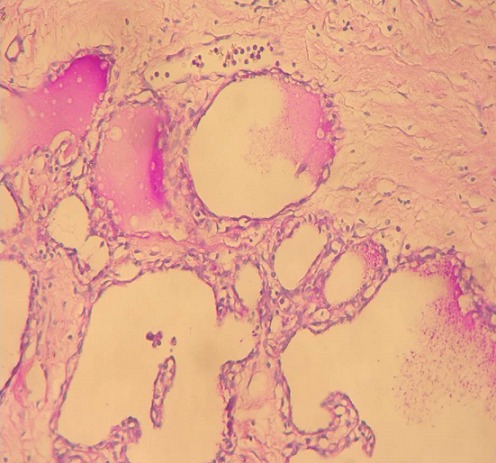
PAS positif (HEx200)

Nous rapportons l'observation d'une patiente âgée de 32 ans, explorée pour des douleurs épigastrique isolées évoluant depuis 3 mois, l'examen clinique et le bilan biologique étaient sans anomalies; l'exploration par échographie et scanner avait conclu a une tumeur compressive et kystique de la queue du pancréas faisant 6 cm de grand axe. la patiente a bénéficié d'une une splénopancréatectomie caudale avec découverte per opératoire d'un nodule friable de 0.5 cm, de siège péri-pylorique. A L'examen anatomopathologique, le nodule péri pylorique mesuraient 0.5 cm, et correspondait à un tissu pancréatique hétérotopique comportant des structures canalaires de taille variée parfois dilatées à revêtement excréto-pancréatique, associés à de discrets foyers d'acini pancréatiques et à un contingent musculaire périphérique (Figure 1). La nature pancréatique a été confirmée par étude immunohistochimique montrant une forte immunoréactivité à la CK19+ et à l'ACE+ avec présence de quelques ilots de cellules neuroendocrines exprimant fortement la synaptophysine (Figure 2, Figure 3) La pièce de splénopancréatectomie caudale renfermait une tumeur de 6x5x3 cm, encapsulée, bien limitée, d'aspect micro kystique, à contenu séreux, sans communication avec les canaux pancréatiques (Figure 4). Elle répondait histologiquement à une formation multi kystique faite de nombreuses cavités, de taille variable, parfois accolées, tapissées par un revêtement épithélial cubique ou aplati, envoyant des micro-projections pseudo- papillaires intraluminales, les cellules étaient munies de noyaux arrondis à chromatine fine, sans atypies, à cytoplasme réduit, chargé de mucines neutres PAS+ et Bleu Alcian (Figure 5, Figure 6). Le diagnostic de cystadénome séreux de la queue du pancréas associé à une hétérotopie pancréatique pylorique a été retenu.

## Discussion

L'hétérotopie pancréatique(HP) est un tissu pancréatique qui n'a pas de rapports anatomiques ni vasculaires avec le pancréas. Il peut se rencontrer dans le tractus gastro-intestinal, plus fréquemment au niveau de l'estomac (25%-38%) de localisation souvent antrale (80%), duodénum (17%-36%) et jéjunum (15%-21%). De rares cas étaient décrits au niveau des voies biliaires, la vésicule biliaire, la rate et le mésentère. Sur le plan anatomopathologique, on peut distinguer 4 sous types; type 1: tissu pancréatique typique, type 2: canaux pancréatiques, type 3: acini, type 4: ilots de Langherans [1, 2]. Elle peut se voir à tout âge avec une légère prédominance masculine. Deux théories sont admises pour expliquer l'histogenèse de l'HP et qui sont la migration soit de tissu pancréatique primitif vers une localisation ectopique ou il devient mature soit de la métaplasie pancréatique de l'endoderme dans la sous muqueuse pendant l'embryogenèse. Typiquement, le pancréas aberrant est bénin et asymptomatique; découvert fortuitement en per opératoire dans 0.5%, comme c'est le cas de notre patiente, lors d'une autopsie ou lors d'une endoscopie digestive. Il apparait sous la forme d'un nodule sous muqueux ombiliqué au centre. Le plus souvent, aucune symptomatologie n'est rapportée [1]. Si la maladie est symptomatique, les manifestations cliniques vont être en rapport avec le siège, la taille, et les phénomènes obstructifs causés par la masse (cholécystite, obstacle biliaire, invagination intestinale’) ou à des saignements intestinaux [1–4]. Les altérations lésionnelles sont les mêmes que celles observées au niveau du pancréas. Il était décrit, dans la littérature, l'association de HP à des lésions digestives, surtout malformatives, à savoir: dilatation kystique du cholédoque, diaphragme duodénal, atrésie de l’œsophage. Nous rapportons, à travers notre observation, l'association d'une HP à un cystadénome séreux du pancréas, constituant elle-même une lésion rare représentant seulement 1 à 2% des tumeurs pancréatiques. Cette association parait, en effet, fortuite [3]. Le diagnostic histopathologique de l'HP est, en général, aisé surtout s'il s'agit de type 1 sinon, comme dans notre observation, on peut recourir à l'immunohistochimie (CK19, synaptophysine, ACE). Le diagnostic différentiel essentiel est l'adénomyome gastrique et seul la présence de pancréas endocrine ou des structures acineuses peut trancher [3, 4]. Le traitement consiste à une résection chirurgicale si l'HP est symptomatique ou si découverte per opératoire pour éviter le risque de dégénérescence, rapportée par quelques auteurs.

## Conclusion

L'hétérotopie pancréatique est une lésion bénigne rare souvent de découverte fortuite, elle est de diagnostic, souvent, facile mais peut dans certains cas prendre l'aspect morphologique d'un adénomyome gastrique. Elle peut être associée à des lésions pancréatiques kystiques. Nous avons porté le diagnostic d'hétérotopie du pancréas devant l'aspect histologique de la lésion qui associait des structures tubuleuses et acinaires, identiques à celles observées dans le pancréas normal.
